# Computed tomography with segmentation and quantification of individual organs in a *D. melanogaster* tumor model

**DOI:** 10.1038/s41598-022-05991-5

**Published:** 2022-02-08

**Authors:** Petter Holland, Eduardo Martin Quintana, Rojyar Khezri, Todd Andrew Schoborg, Tor Erik Rusten

**Affiliations:** 1 Centre for Cancer Cell Reprogramming, Institute of Clinical Medicine, Faculty of Medicine, University of Oslo, Oslo, Norway; 2 Department of Molecular Cell Biology, Institute for Cancer Research, Oslo University Hospital, Ullernchaussen, 0379 Oslo, Norway; 3 Cell Biology and Physiology Center, National Heart, Lung and Blood Institute, National Institutes of Health, Bethesda, MD 20892 USA

**Keywords:** Imaging, Cancer, Cancer models

## Abstract

*Drosophila melanogaster* tumor models are growing in popularity, driven by the high degree of genetic as well as functional conservation to humans. The most common method to measure the effects of a tumor on distant organs of a human cancer patient is to use computed tomography (CT), often used in diagnosing cachexia, a debilitating cancer-induced syndrome most visibly characterized by loss of muscle mass. Successful application of high resolution micro-CT scanning of *D. melanogaster* was recently reported and we here present the segmentation of all visible larval organs at several stages of tumor development. We previously showed the strong expected reduction in muscle mass as the tumor develops, and we here report a surprisingly strong reduction also in gut and Malpighian tubules (kidney) volume. Time-point of tumor development was found to have a stronger correlation to cachectic organ volume loss than tumor volume, giving support to the previously proposed idea that tumor size does not directly determine degree of cachexia.

## Introduction

*Drosophila melanogaster* with genetic programs causing predictable tumor growth is a cancer model that is growing in popularity and has recently been used to study how tumors interact with the microenvironment^1^, systemic insulin signaling^2,3^ and systemic metabolism^4^. Several oncogenes and tumors suppressors common in human cancers are demonstrated to be conserved in the fly and when genetically manipulated, can contribute to tumor development^5^. The commonality to human cancer seen together with the simple genetics and short time-spans required to perform experiments makes it likely that there will be a growing interest in *D. melanogaster* as a cancer model in the future.

There is a rapidly growing body of literature describing interactions between a growing tumor and neighboring cells and the immune system in the microenvironment^6^, but considerably less knowledge about long-range systemic consequences of a tumor. Tumor presence probably affects all the organs of the tumor’s host in vivo to some degree, but the most dramatic presentation is cancer-induced cachexia, most visibly characterized by rapid loss of muscle volume and adipose tissue^7^. Cachexia also affects the brain, liver and the gut, but the mechanisms and consequences on these organs are less studied.

We have recently described a role for autophagy in tumor-induced muscle wasting^8^ and for that study we performed micro computed tomography (CT) of larvae to obtain a measurement of tumor and muscle volumes at several stages of tumor development in the intact whole larvae. Here, we present the segmentation, quantification and extended analysis of all the other organs visible in the CT scans of these larvae. We find a surprisingly strong reduction in gut size and effects on the Malpighian tubules as cachexia progresses. We also compare the correlation of the measured cachexia outcomes with either tumor size or time with tumor presence and find support for earlier claims^3,9^ that degree of cachexia does not appear to be directly determined by tumor size.

## Results

### Micro-CT scanning and segmentation of organs in *D. melanogaster* larvae with tumors

**Figure 1:**
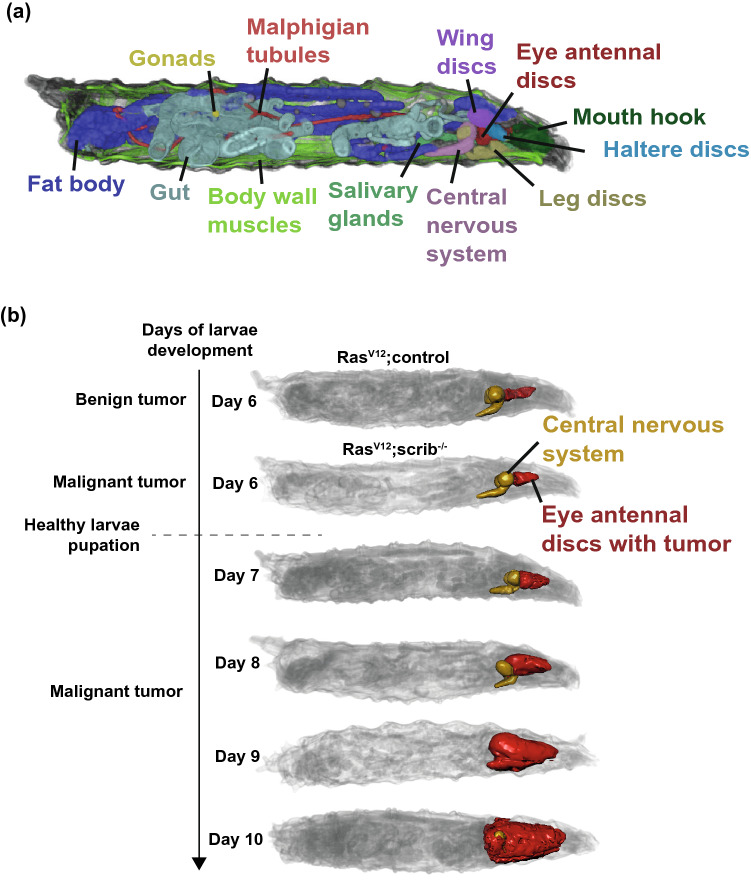
Segmentation and quantification of organs in a *D. melanogaster* tumor model. (**a**) The main organs of the larvae indicated in different colors. (**b**) An illustration of the brain and eye antennal discs (EAD) where the tumor is growing in *Ras*^*V12*^*;control* and *Ras*^*V12*^*;scrib*^*−/−*^ larvae at several time-points. *Ras*^*V12*^*,scrib*^*−/−*^ larvae have an exponentially growing tumor in the EAD that becomes visible around day 6. In healthy larvae, pupation starts around day 6, but when there is a tumor, pupation is delayed. Quantification of EAD volumes of these larvae were reported in Khezri et al.^8^. Shown images are of representative individual larvae.

We induced tumors in larvae by stochastic activation of the oncogene *Ras*^*V12*^ and knockout of the tumor suppressor *scribble* (maintains cell polarity) in cells of the eye antennal disc (EAD) during development. The individual transformed cells will grow in an environment of normal cells, resembling normal tumor growth. Three-dimensional scans of the fixed intact larvae were obtained through high resolution micro-CT (Fig. 1a). To measure the gradual changes to the host with an increasing tumor burden over time, we prepared larvae with tumors at day 6,7,8,9 and 10 since the beginning of larval development (Fig. 1b). Along with these time-points of larvae with *Ras*^*V12*^*;scribble*^*−/−*^ tumors, control larvae with *Ras*^*V12*^*;control* benign tumors at day 6 were also scanned. From our previous work^8^ we know that the *Ras*^*V12*^*,scrib*^*−/−*^ larvae at day 6 are in a pre-cachectic state, showing systemic metabolic effects of the small malignant tumor presence, but no obvious muscle wasting, while at day 8 the larvae are cachectic, characterized by reduced muscle volume. To define which other organs are similarly affected by the expanding tumor, but minimizing other confounding factors such reduced feeding (larval feeding is normal until day 8 of development with tumor^8^), we focused on the comparison of the day 6 to day 8 *Ras*^*V12*^*,scrib*^*−/−*^ larvae throughout this analysis to define which organs are changed due to cachexia.

The micro-CT scans were segmented in DragonFly^10^, where the different organs were differentiated and segmented manually by the position, shape and tissue density. In Fig. 1a we present the segmentation of all CT-visible larval *D. melanogaster* organs, given different colors. Two organs could not be reliably segmented because their density is too similar to surrounding tissues, meaning their size and position could not be reliably defined—the heart and lymph gland. We previously presented the quantification of how the EAD volume increases exponentially as the tumor grows, expanding sevenfold between day 6 and day 10 of larval development^8^. The EAD is positioned next to the central nervous system (CNS) in the larvae and the tumor envelops the brain and ventral nerve cord as it grows. While the CNS of the larvae is most likely functionally affected by being almost enveloped by a tumor at later stages of tumor development, we find that the volume of the CNS is not affected (Supplementary Fig. 1a). The salivary glands and mouth hook are also positioned in the anterior of the larvae close to the tumor, but we did not detect any significant changes to the volumes of these organs as a consequence of the tumor (Supplementary Fig. 1b–d). Quantification of organ volumes from smaller discs in the anterior of the larvae was increasingly challenging with a bigger tumor. Leg and haltere disc volumes were not possible to determine after day 6 and wing disc volumes were not determined at day 10 (Supplementary Fig. 2a,b).

### Micro-CT quantification of posterior body wall muscles is a novel gold-standard of fly cachexia measurement

**Figure 2:**
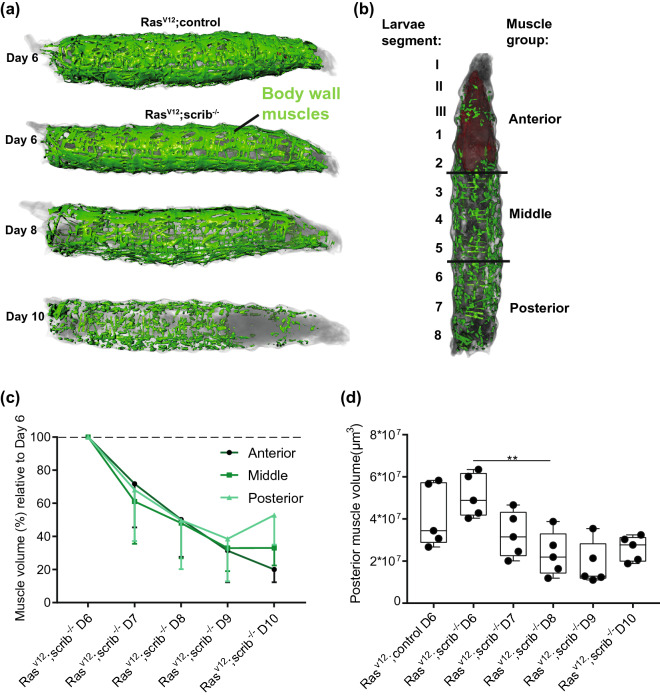
Posterior muscle volumes are strongly reduced from day 6 to day 8 of tumor development. (**a**) Illustration of body-wall muscles in a control larvae or with a tumor at several time-points. (**b**) Illustration of how we divide the larval muscles into groups to analyze how muscle position relative to the tumor affects the muscles. The tumor is growing in the anterior of the animals. (**c**) Summed volumes of the different muscle groups, shown relative to the day 6 measurement to illustrate how the muscle volumes change in different parts of the animal due to the anterior tumor. (**d**) Posterior muscle volumes shown for all time-points. The indicated statistical test is one-way ANOVA with a Turkeys’s multiple comparisons test. **p < 0.01. Shown images are of representative larvae. Quantifications in (**c**) and (**d**) are of n = 5 individual larvae.

We previously reported the quantification of total muscle volume in these larvae^8^. From the scans of the day 10 larvae, it was apparent that the muscles were often completely missing from the anterior of the larvae, close to the tumor (see Fig. 2a, day 10). If the tumor is only affecting nearby muscles, this would not be cachexia, which is defined as a systemic syndrome mediated through the circulation of the animal and should not require close contact. Therefore, it was important to establish to what degree muscles in the posterior are affected by the tumor growing in the anterior of the animal. We divided the larva body wall muscles in 3 groups (Fig. 2b) and added together the muscle volume measurements within each group to determine the relative changes in volume as the tumor grows (Fig. 2c). All three groups show a steady decline in muscle volume with a consistent ~ 50% loss of muscle volume from day 6 to day 8. From day 8 to day 10 there is a divergence between the muscle groups with the anterior muscles continuing to decline in volume compared to the other parts of the larvae. We conclude that the muscle loss in posterior muscles from day 6 to day 8 is a long-range effect of the tumor mediated through the circulation of the animal, consistent with cachexia (Fig. 2d). The changes to the anterior muscles from day 8 to day 10 may be an additional more localized effect from short-range signaling from the tumor or by the tumor directly pushing on the muscles.

### Foregut, midgut and Malpighian tubules organ volumes are reduced in cachectic animals

**Figure 3:**
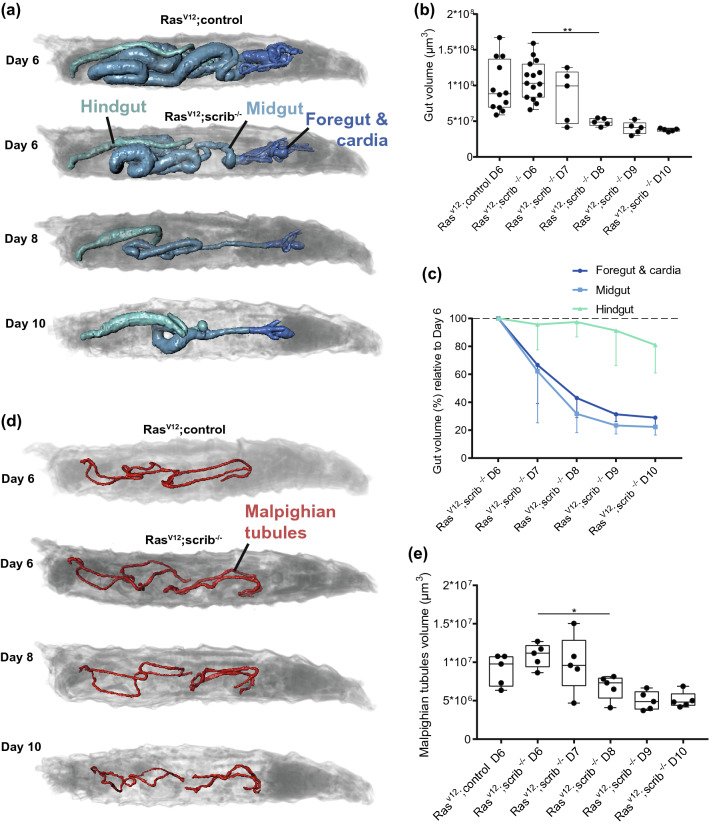
The foregut and cardia, midgut and Malpighian tubules all demonstrate reduced organ volume during cachexia. (**a**) Illustration of the gut segments in a control larvae or with a tumor at several time-points. Foregut (esophagus) and cardia are measured as one unit and shown as dark blue, midgut (small intestine) in light blue and hindgut (large intestine) is shown in teal. (**b**) Quantification of total gut volume for all samples. (**c**) Volumes of the three distinct parts of the gut quantified separately and shown relative to the day 6 measurements. (**d**) Illustration of Malpighian tubules (kidneys). (**e**) Malpighian tubules volumes shown for all samples. The indicated statistical tests are one-way ANOVA with a Turkeys’s multiple comparisons test. *p < 0.05. **p < 0.01. Shown images are of representative larvae. Quantifications in (**b**) have n = 15 for *Ras*^*V12*^*;control* D6 and *Ras*^*V12*^*;scrib*^*−/−*^ D6 and n = 5 for the remaining samples of that graph. Quantifications in (**c**) and (**e**) are of n = 5 larvae.

The fat body of the *D. melanogaster* larva is functionally both adipose and liver tissue and loss of adipose tissue is commonly seen in human cachexia. We have previously described morphological changes to the lipid droplets in the fat body in this model as the tumor grows, but we did not detect a loss of fat body volume in these larvae^8^ (Supplementary Fig. 2c). A recently discovered feature of cachexia, gut barrier dysfunction, has only been described in a mouse model^11^. In *D. melanogaster*, there are three functionally and morphologically distinct parts of the gut, the foregut (esophagus) and cardia, midgut (small intestine) and hindgut (large intestine). We quantified the volumes of the parts of the gut in our model over time and found surprisingly large morphometric changes to the gut as the tumor grows (Fig. 3a,b).The foregut and cardia as well as midgut decrease more than 50% in volume from day 6 to day 8 while the hindgut is more stable (Fig. 3c).

Also the Malpighian tubules, which are functionally equivalent to kidneys and attached to the gut, are affected by the tumor and have a significantly reduced volume between day 6 and 8 (Fig. 3d,e). Both the gut and the Malpighian tubules are tubular structures and a change in the total volume can be either due to a change in the thickness or the length of the tube. We measured these parameters independently and found that for the gut, the change in volume is because of a reduced length of the foregut and midgut (Supplementary Fig. 3a,b) while for the Malpighian tubules the organ volume is less because the thickness of the tubes is reduced (Supplementary Fig. 3c,d). From the size of the gonads of the scanned larvae we could also determine the sex of the animal. Unfortunately, we did not pre-select larvae for scanning based on sex so the groups of males and females are unequal and not sufficiently large to draw any conclusions about possible differences in organ volumes due to the sex. We did not observe any obvious strong difference in cachectic organ wasting due to sex differences in the samples we could compare (Supplementary Fig. 4a–d). Additional studies will be required to conclude on this matter.

### Cachectic organ volume changes are more strongly correlated to time with tumor presence than with tumor size

**Figure 4:**
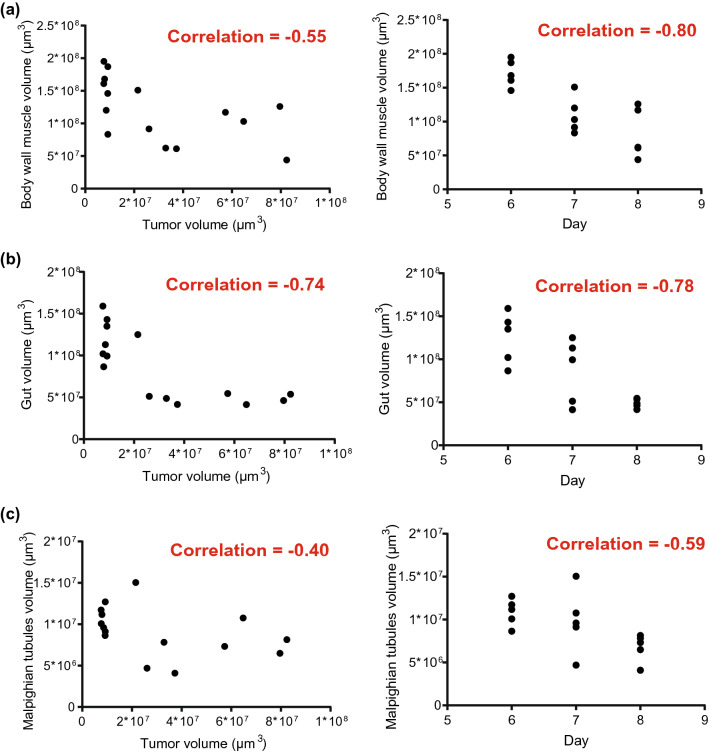
The correlation of organ volume loss is stronger to time with tumor than it is with tumor size. (**a**) Whole-animal body wall muscle volumes compared to tumor volume or time-point. (**b**) Whole gut volume compared to tumor volume or time-point. (**c**) Malpighian tubules volume compared to tumor volume or time-point. For all plots, the red line indicates a linear regression fit of all datapoints. The Pearson correlation of the x- and y-axis values is shown in red in each graph. All panels are of n = 15 larvae in total. In panels where the x-axis shows days, each day has n = 5 larvae.

Having matched measurements of tumor volume and organ sizes from individual larvae, we explored how strong the correlation is between tumor volume and organ volumes of the same animal. If the amount of cachectic organ volume loss is a direct causal effect of tumor size, we would expect the correlation to be stronger between tumor volume and organ volume compared to time point and organ volume. We found that the opposite is true for all three organs where we found a significant reduction in size from day 6 to day 8 (Fig. 4a–c). The egg that eventually becomes these larvae can be laid at any time during a 1-day window, meaning that the time-point measurements are grouped together compared to the tumor size measurements, which are continuous. This difference makes it difficult to draw strong conclusions based on the comparisons of correlations from these two types of observations. However, we note that the differences we see in correlations (Fig. 4a–c) are in line with previously published data from other model organisms^3,9,12^ giving support to the idea of tumor size not directly being causal in determining the amount or rate of organ wasting.

## Discussion

Obtaining reliable measurements of muscle volume in *D. melanogaster* tumor models is a significant technical challenge because of how sensitive the measurements are to stretching of the small pieces of tissue during sample preparation. To get around this problem, a surrogate read-out of fly cachexia has been used in multiple studies; an accumulation of liquid in the animal causing a “bloated” phenotype which can easily be screened for. This is believed to be a secondary effect of cachexia, but there is recent data suggesting the bloating may be a direct consequence of tumor-induced accumulation of trehalose in circulation^13^, and therefore not necessarily related to cachexia. Furthermore, although insulin resistance and reduced glucose tolerance are often seen also in human cachexia, oedema (equivalent to bloating in humans) is not seen in cachectic cancer patients^14^. Whole-body CT scanning is the gold-standard for diagnosing and quantifying human cachexia^15^ and we propose that also for smaller model organisms like flies, micro-CT measurement of intact animals should be the reference method to quantify cancer-induced cachexia. Obtaining these measurements is resource-intensive, but should be performed to validate proposed surrogate measurement of fly cachexia.

The strongest effect on the organism from the growing tumor is the dramatic shrinkage of anterior parts of the gut. It is known from mice that gut barrier dysfunction can occur with cachexia, leading to systemic inflammation^11^. Cachexia in humans is also known to correlate with systemic inflammation, leading to several clinical trials attempting to eliminate cachexia by blocking inflammation. These trials concluded with negative results^16,17^, and a possible explanation to this is that inflammation may be a consequence of cachexia, rather than a cause of it. These observations, seen together with the strong decrease in gut volume that we observe in our model, suggests that more focus should be placed on the consequences on the gut in studies of cachexia. We also see significant morphological changes to the Malpighian tubules as cachexia develops. Because the Malpighian tubules are functionally equivalent to kidneys, a loss of Malpighian tubule function could explain the observed bloating in cachectic larvae, caused by a loss of osmotic control in the animal. However, there is no strong link between human cachexia and loss of kidney function, so this phenotype would be unique to the fly model.

For human cancer patients, cachexia is observed relatively late in the disease progression, when there is a large tumor burden. It is common for cachectic patients to improve if their tumor burden is reduced through surgery or chemotherapy, but the link between tumor burden and likelihood of developing cachexia is complicated. Also in model organisms the degree to which the development and severity of cachexia is directly determined by tumor size is still debated^18^. Our micro-CT dataset of tumor and cachectic tissue volume measurements from the same intact animals at several stages of tumor development and cachexia is a valuable contribution to the debate about the link between tumor size and cachexia. The stronger correlation we see of muscle wasting with time rather than with tumor size gives support to previous claims that cachexia is not directly determined by tumor size^3,9^. If time with tumor presence is a stronger determinant than tumor size, this could help explain some of the conflicting observations of previous studies because tumor size usually increases with time. One possible mechanism to explain this would be if cachexia was induced by a tumor-secreted factor and gradually progresses over time at a steady rate that is not influenced by the amounts of the secreted factor.

## Methods

### Fly husbandry

Stocks and crosses were kept at 25 °C on standard potato mash fly food containing 32.7 g dried potato powder, 60 g sucrose, 27.3 g dry yeast, 7.3 g agar, 4.55 ml propionic acid, and 2 g nipagin per liter, resulting in a final concentration of 15.3 g l^−1^ protein and 6 g l^−1^ sugar.

### Fly genetics

*y,w,ey-FLP; Act* > *y*^+^ > *Gal4, UAS-GFP; Frt82B, tub-GAL80* virgin females were crossed with *y,w; UAS-Ras*^*V12*^*; Frt82B/SM6-TM6* males (for *Ras*^*V12*^*;control*) or *y,w; UAS-Ras*^*V12*^*; Frt82B,scrib*^*−/−*^*//SM6-TM6* males (for *Ras*^*V12*^*; scrib*^*−/−*^). Non-tubby offspring larvae develop aggressive tumors in the eye disc because of the combined loss of scribble (tumor suppressor) and the overexpression of Ras^V12^ (oncogene).

*Ras*^*V12*^*;control: y,w,ey-FLP/y,w;UAS-Ras*^*V12*^*/act > y*^*+*^*>GAL4, UAS-GFP; Frt82B/Frt82B, tub-GAL80*.

*Ras*^*V12*^*;scrib*^*−/−*^*: y,w,ey-FLP/y,w;UAS-Ras*^*V12*^*/act > y*^*+*^* > GAL4, UAS-GFP; Frt82B, scrib*^*1*^*/Frt82B, tub-GAL80*.

Healthy control *D. melanogaster* larvae will start pupation around day 6 of development. Only animals with a tumor, threatening infection or hormonal deficiencies will delay pupation and therefore remain as larvae after day 6 of development. In Khezri et al.^8^ we investigated the muscles of two different fly strains with hormonal deficiencies at day 10 and found no changes in muscle volume due to the delayed pupation alone.

### Ethics approval

The experiments with Drosophila melanogaster were performed in accordance with The Norwegian Department of Health’s guidelines and approvals for transgenic animals and facilities #20/26339-11.

### µ-CT

#### Labeling and mounting

Larvae were collected and transferred to a 1.5 ml Eppendorf tube with 1 ml of 0.5% (v/v) PBST (Phosphate Buffered Saline, Triton-X 100) and incubated for 5 min at room temperature. Larvae were then fixed in 1 ml Bouin’s solution (Sigma Aldrich, St. Lous, MO) for 2 h at room temperature. A microdissection needle was then used to poke a small hole in the cuticle at both the anterior and posterior ends of each larva, carefully avoiding any underlying soft tissue. Larvae were incubated again in fresh Bouin’s solution for 16–22 h at room temperature. Larvae were then washed 3 times for 30 min in 1 ml of µ-CT Wash Buffer (0.1 M Na2HPO4, 1.8% sucrose) and stained with 1 ml of a 0.1 N solution of I2KI (Lugol’s solution) for 1–2 days at room temperature. Larvae were then washed twice in ultrapure water and stored at room temperature. Individual larvae were mounted for scanning using a P10 pipette tip filled with water, wedged inside a small piece of plastic capillary tube that fits tightly in a custom-made brass holder. A dulled 20-gauge needle was used to gently push the larva down in the pipette tip until it became wedged along the wall.

#### Scanning

Larvae were scanned at the Cell Biology and Physiology Center, National Heart, Lung and Blood Institute, using a SkyScan 1172 desktop scanner controlled by Skyscan software (Bruker) operating on a Dell workstation computer (Intel Xeon X5690 processor @ 3.47 GHz (12 CPUs), 50 GB RAM and an NVIDIA Quadro 5000 (4 GB available graphics memory) GPU). Data sets were acquired with the X-Ray source at 40 kilovolts (kV), 110 microamps (μA) and 4 watts (W) of power. A Hamamatsu 10Mp camera with an 11.54 μm pixel size coupled to a scintillator was used to convert X-rays to photons. Medium camera settings at an image pixel size of 4.5 μm were used for around 20 min’ scans, acquiring about 300 projection images with an effective resolution of 25–50 μm. Random movement was set to 10 and frame averaging ranged from 4 to 8.

### Reconstruction

Tomograms were generated using NRecon software (Bruker MicroCT, v1.7.0.4) base on Feldkamp algorithm, resulting in images with an isotropic voxel size of 60.42 μm^3^. This reconstruction software uses reference scans to correct for sample drift in the projection images, followed by an iterative application of misalignment and ring artifact reduction algorithms to generate the highest quality images possible^19^.

### Image analysis

Tomograms were visualized and analyzed using Dragonfly software (v4.1, Object Research Systems (ORS) Inc, Montreal, Canada, 2019; software available at http://www.theobjects.com/dragonfly) operating on a Dell Precision T7600 workstation (Intel® Xeon^®^ CPU E5-2620 @ 2.00 GHz, 32 GB RAM, 64-bit Operating System, 3 GB NVIDIA Quadro K4000 GPU) in the form of a 16-bit grayscale bitmap. Segmentation of anatomical structures into individual regions of interest (ROIs) was performed manually using the 3D paintbrush function through the z-stack sections on the axial view based on Volker Hartenstein’s Atlas of Drosophila Development^20^. To eliminate human bias during segmentation, a threshold base value that encompassed entirely each structure of interest was selected. This threshold value was then applied to all images and used for segmentation of tissues into ROIs. Due to differences in contrast between samples (as a result of X-Ray beam fluctuations) small adjustments of this threshold value were made, when necessary, to precisely delineate the tissue boundaries. ROIs were then rendered to meshes and smoothed using Laplacian smoothing algorithm to create more accurate representations of the structure. The built-in exporting 2D images tool and 3D movie maker were used to generate all larvae pictures.

### Length measurements

Micro-CT Z-stacks were opened using FIJI with a pixel width, pixel height and voxel depth of 3.92 μm; and loaded into the Simple Neurite Tracer plugin^21^. The gut length was measured from just after the cardia to the end of the hindgut using the center of the lumen as trace reference. Since this tool trace objects of high intensity and the lumen intensity is low, the lookup table was inverted when the gut was analyzed. The Simple Neurite Tracer plugin was also used to analyze the anterior and posterior structures of the Malpighian tubules separately and summed to calculate the total Malpighian tubules length.

### Statistical analysis

Quantitative analysis for each organ is presented as mean ± SD in scatter boxplot or line graph. A P value < 0.05 was considered statistically significant for all datasets. Statistical significance was determined by using one-way ANOVA followed by Turkeys’s multiple comparisons test for multiple samples assuming a normal distribution of the data. Adjusted P values and sample size are indicated in the figure legends. Linear regression analysis is presented with a Pearson correlation coefficient and p-value for the variable in linear regression fit. All analysis and graphs were generated using GraphPad Prism 8.

## Supplementary Information

Supplementary Figures.
