# Supplementary material for: Computed tomography with segmentation and quantification of individual organs in a D. melanogaster tumor model
Source: Sci Rep. 2022 Feb 8;12:2056. doi: 10.1038/s41598-022-05991-5 (PMC8825794; doi:10.1038/s41598-022-05991-5)
Supplement: Supplementary file 1 — Supplementary Figures. [file 41598_2022_5991_MOESM1_ESM.docx]

**
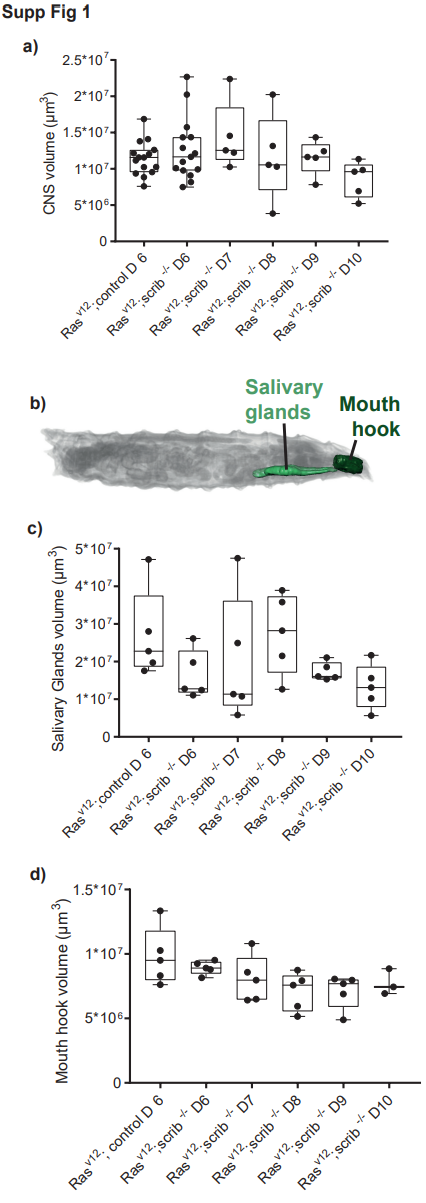
**

**Supplementary Figure 1: Anterior organs that were segmented and quantified, but where organ volume is not affected by the tumor.** a) Central nervous system (CNS) organ volumes. See Figure 1 b for a visual demonstration of the CNS in these larvae. b) Illustration of the salivary glands and mouth hook, with organ volume quantifications shown in c (salivary glands) and d (mouth hook). Shown quantifications are of n=5 larvae with the exception of *Ras^V12^;control* D6 and *Ras^V12^;scrib^-/-^* D6 in a) which has n=15 and *Ras^V12^;scrib^-/-^* D10^-/-^ in d) which has n=3 because the mouth hook was overgrown by the tumor in some animals at day 10.

**
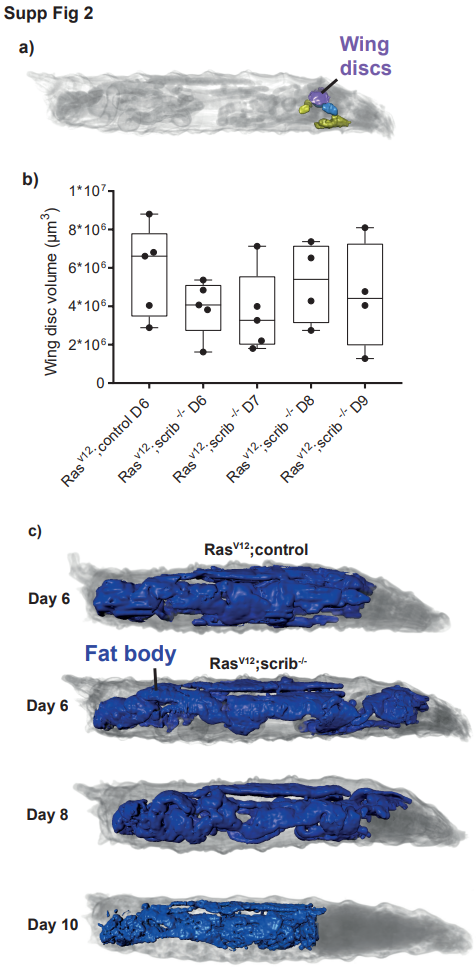
**

**Supplementary Figure 2: Total fat body volume is not affected by the tumor.** a) Illustration of the smaller anterior discs. b) Wing disc organ volumes quantified for all samples except day 10 which could not be obtained because the tumor invades surrounding tissues at day 10, making it impossible to reliably segment several nearby discs. This problem also affects the smaller leg discs (yellow in a) and halter discs (blue in a), which are difficult to reliably segment at several time-points. c) Illustration of the fat body with or without a tumor. Quantification of fat body volume was presented in Khezri et al (EMBO J, 2021). n=5 for shown quantifications except for b) *Ras^V12^;scrib^-/-^* D8 and D9 which has n=4.

**
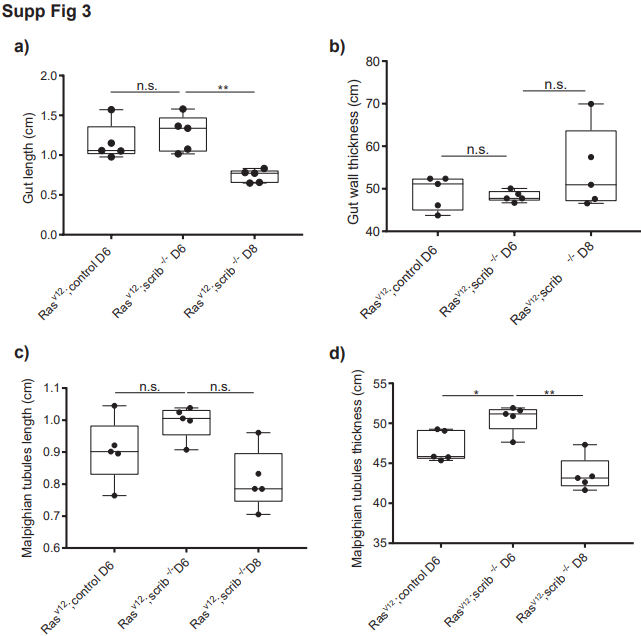
**

**Supplementary Figure 3: Tumor presence affects the volume of the gut or Malpighian tubules in different ways.** a) Length of the gut. b) Gut wall thickness. c) Malpighian tubules length. d) Malpighian tubules thickness. The indicated statistical tests are one-way ANOVA with a Turkeys’s multiple comparisons test. * = p < 0.05. ** = p < 0.01. n=5 for all shown quantifications.


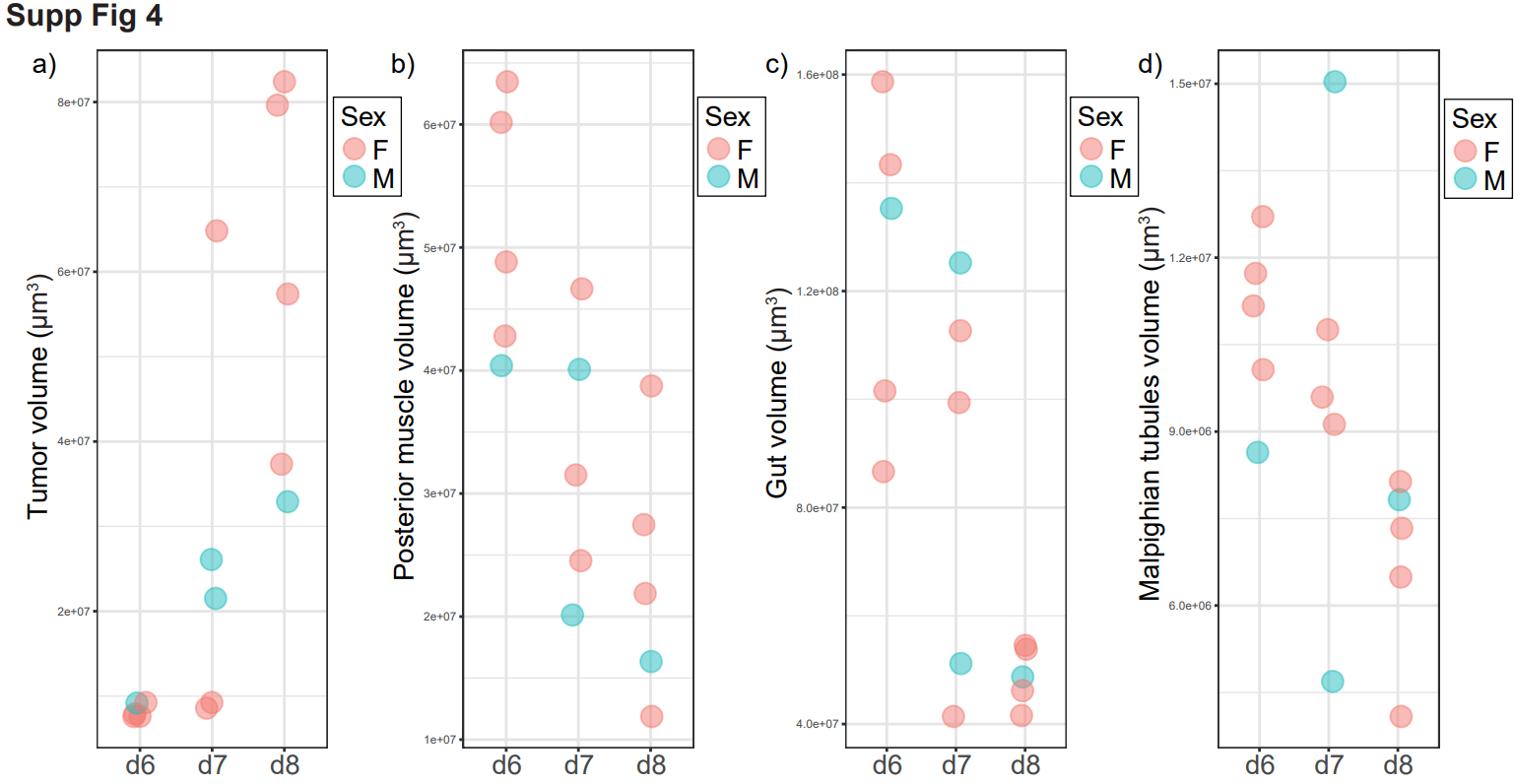


**Supplementary Figure 4: Tumor and cachectic organ volumes, differentiated by the sex of the animals.** Tumor (a), posterior muscle (b), gut (c), Malpighian tubules (d) organ volumes, shown for pre-cachectic (day 6) to cachectic (day 8). The measurements above are also shown in the following figures, without sex information: a) in Khezri et al, EMBO J, 2021. b) Figure 2 d, c) Figure 3 b, d) Figure 3 e. n = 5 for all measurements above, with 1 male and 4 females in day 6 measurements, 2 males and 3 females in day 7 measurements, 1 male and 4 females in day 8 measurements.
